# A Recombinant Mosaic HAs Influenza Vaccine Elicits Broad-Spectrum Immune Response and Protection of Influenza a Viruses

**DOI:** 10.3390/vaccines12091008

**Published:** 2024-09-02

**Authors:** Xuejie Liu, Chuming Luo, Zhuolin Yang, Tianyi Zhao, Lifang Yuan, Qian Xie, Qijun Liao, Xinzhong Liao, Liangliang Wang, Jianhui Yuan, Nan Wu, Caijun Sun, Huacheng Yan, Huanle Luo, Yuelong Shu

**Affiliations:** 1 School of Public Health (Shenzhen), Shenzhen Campus of Sun Yat-sen University, Shenzhen 518107, China; liuxj89@mail2.sysu.edu.cn (X.L.);; 2 National Institutes for Food and Drug Control, Chinese Academy of Medical Sciences & Peking Union Medical College, Beijing 100730, China; 3 Shenzhen Nanshan Center for Disease Control and Prevention, Shenzhen 518054, China; 4 Center for Disease Control and Prevention of Southern Military Theatre, Guangzhou 510610, China; 5 Key Laboratory of Pathogen Infection Prevention and Control (Peking Union Medical College, Ministry of Education), State Key Laboratory of Respiratory Health and Multimorbidity, National Institute of Pathogen Biology of Chinese Academy of Medical Science (CAMS)/Peking Union Medical College (PUMC), Beijing 100730, China

**Keywords:** influenza, hemagglutinin, mosaic, recombinant protein, universal vaccine

## Abstract

The annual co-circulation of two influenza A subtypes, H1N1 and H3N2, viruses in humans poses significant public health threats worldwide. However, the continuous antigenic drift and shift of influenza viruses limited the effectiveness of current seasonal influenza vaccines, necessitating the development of new vaccines against both seasonal and pandemic viruses. One potential solution to this challenge is to improve inactivated vaccines by including multiple T-cell epitopes. In this study, we designed stabilized trimeric recombinant mosaic HA proteins named HAm, which contain the most potential HA T-cell epitopes of seasonal influenza A virus. We further evaluated the antigenicity, hemagglutinin activity, and structural integrity of HAm and compared its immunogenicity and efficacy to a commercial quadrivalent inactivated influenza vaccine (QIV) in mice. Our results demonstrated that the HAm vaccine was able to induce broadly cross-reactive antibodies and T-cell responses against homologous, heterologous, and heterosubtypic influenza-naive mice. Additionally, the HAm antigens outperformed QIV vaccine antigens by eliciting protective antibodies against panels of antigenically drifted influenza vaccine strains from 2009 to 2024 and protecting against ancestral viruses’ lethal challenge. These results suggest that the HAm vaccine is a promising potential candidate for future universal seasonal and pandemic influenza vaccine development.

## 1. Introduction

Influenza is a highly contagious viral disease that poses a significant public health risk, even with the availability of antivirals and licensed vaccines [1]. The surface of the influenza virion is studded with two glycoproteins: hemagglutinin (HA) and neuraminidase (NA) [2]. HA is the most crucial antigen in influenza vaccines, triggering immune response-neutralizing antibodies against influenza virus. As a result, it is the key or sole antigen in all commercial influenza vaccines [3]. However, the immunodominant HA is prone to frequent antigenic shift and drift, resulting in the influenza viruses evading adaptive immune responses. This often leads to a mismatch between the vaccine and circulating strain, resulting in suboptimal vaccine effectiveness [4,5]. Therefore, the development of a novel universal vaccine with broad protection is urgently needed.

Over the past few decades, influenza vaccine practices and production methods have improved greatly [6,7,8]. While current egg-based inactivated influenza vaccines (IIV) have performed well in preventing infection and eliciting protective antibody responses [9,10,11], they still have several problematic features that cannot be ignored. For instance, they rely on an adequate supply of eggs, and their extended production time may not be able to respond quickly to pandemic threats [12]. Moreover, people with egg allergies may be at increased risk of reacting to egg-based vaccines [13]. Most importantly, some influenza viruses cannot be cultured in eggs or undergo egg-adapted mutations during culture [14,15], which can alter the immunogenicity of HA or mask critical antigenic sites on HA proteins [16]. Given the disadvantages in the production and immunogenicity of egg-based vaccines, recent efforts have been made to develop new production strategies or platforms [17,18]. One of the licensed vaccine production platforms that has become available recently is based on a baculovirus expression system [18,19]. This system allows for the massive and rapid production of recombinant HA proteins and is less likely to cause HA mutations. The individual HA proteins are expressed, purified, and combined into multivalent vaccines without interference from non-HA proteins of other sources [16]. The recombinant HA proteins expressed and purified from insect cells are much easier than the purification of HA proteins from virions, which is difficult [18,20]. The complete antigenicity and immunogenicity of HA require a final folding step accompanying its trimerization, which [21] is essential for the induction of protective immune responses [22]. Previously, a trimeric variant from yeast transcription factor, GCN4-pII, termed the leucine zipper, was used to produce stable recombinant trimeric HA, and these constructs improved the antibody level after vaccination [23].

Previously, our group used the mosaic strategy to generate candidate vaccine protein cocktails that optimize the coverage of potential T-cell epitopes in a diverse set of proteins using a small set of mosaic proteins [24]. This algorithm has been successfully applied to develop influenza vaccine candidates and has demonstrated promising results in vivo [25,26]. In consideration of this background, we previously employed the VLP vaccine platform to evaluate the feasibility of mosaic sequences as vaccine antigens, with results indicating their potential for universal vaccine development [27]. Building upon our previous research, the current study employs an alternative mosaic HA antigen expressed using a protein-based vaccine platform and conducts comparative assessments with commercially available vaccines. In this study, we report the development and characterization of HAm, another mosaic HA antigen that contains most potential CD4+ T-cell epitopes, which promotes the humoral and cellular immune responses for broad protection and reduces viral replication immediately after infection. After two doses of HAm vaccination, a strong cross-reactive antibody response was observed in mice, with HAI titer results showing higher than 40 against all last decade’s influenza vaccine strains, while the QIV elicited limited cross-HAI antibody levels. Moreover, HAm vaccination induced CD4+ and CD8+ T cell responses that cleared the virus from infected tissue. These data demonstrate that HAm, expressed by the baculovirus expression system, showed the potential to produce the influenza A virus vaccine in a low-cost way, and it can elicit broad protection against ancestral viruses’ lethal challenge as well as induce broader immunity than QIV. Therefore, the HAm might be a promising universal vaccine candidate against the influenza A virus’ antigenic drift and shift in the future.

## 2. Materials and Methods

### 2.1. Study Design

The main objective of this study was to investigate the immune response and protective efficacy of a recombinant mosaic HAs vaccine against influenza virus in mice. In this study, we used 5 mice per group to assess the immunogenicity of the candidate influenza vaccine. The sample sizes are based on preliminary power calculations, taking into account result variations, aiming to detect biologically significant differences between groups and the statistical power while minimizing the number of animals used. Our preliminary experiments indicated that the standard deviation of immune responses (e.g., HAI results) was approximately 11%, with an effect size greater than 20%. We assumed a significance level of 0.05 and a target power of 80% for our calculations. To evaluate both antibody and T-cell responses, mice were randomly assigned to groups consisting of five individuals. For the challenge experiment, groups of eight mice were challenged, and then the vaccine’s protective effects were assessed based on parameters such as body weight, tissue viral titer, and lung pathology. All mice used in this study were 6-week-old female BALB/c mice.

### 2.2. Ethics Statement

This research strictly followed the guidelines outlined in the Guide for the Care and Use of Laboratory Animals by the Ministry of Science and Technology of the People’s Republic of China. Approval for all experiments was obtained from the Institutional Animal Care and Use Committee and Institutional Review Board at Sun Yat-sen University (approval number: 2022-B013). Experiments involving live viruses and animals were performed in biosafety level 2 (BSL2) animal facilities equipped with negative-pressure isolators with HEPA filters at the Center for Disease Control and Prevention of Southern Military Theatre, People’s Liberation Army.

### 2.3. Mosaic HA Sequences Design 

HA amino acid sequences for human (2009 to 2021) influenza A viruses (H1N1 and H3N2) were obtained from the GISAID and NCBI databases. To avoid redundancy, BioAider and Bioedit were used to remove repetitive and low-quality sequences as well as laboratory strains [27]. The full-length sequences were aligned using MAFFT, and a genetic algorithm was employed to optimize each population. New recombinants were generated, and their epitope coverage was calculated and tested. Two recombinant mosaic HA sequences were obtained, as described previously [24], instead of the epitope length being set to “12”, for covering more CD4+ Th cell epitopes. The mosaic HA sequences were reverse translated and optimized for mammalian, including codon usage and RNA optimization. These optimized sequences were termed computationally optimized broadly reactive antigens (H1m and H3m, Data File S1). 

### 2.4. Gene Cloning, Protein Expression, and Purification

The mosaic sequences of our design were synthesized (Sangon Biotech, Shanghai, China) and cloned into the donor vector pFastBac-Dual between EcoRI and HindIII restriction sites. Successfully cloned recombinant bacmids were purified and used to transfect Sf9 cells. Briefly, a single verified colony of DH10Bac™ *E. coli* (Thermofisher, Waltham, MA, USA) transformed with the designed clone was grown in Luria Bertani (LB) broth overnight at 37 °C to harvest recombinant bacmids. The Sf9 cells were then transfected to culture P0 recombinant baculovirus, amplified, and cultured for P1 and P2 recombinant baculovirus. The supernatant over the cells was harvested and used to assay for protein expression by Western blotting using an anti-hemagglutinin antibody (GTX127357, GTX127363; Genetex, Irvine, CA, USA). Confirmed mosaic HA-expressing Sf9 cells’ supernatants were harvested to continue culture P4 recombinant baculovirus. The supernatant was filtered through a 0.22 μm filter (Millipore, Burlington, MA, USA). HisTrap™ HP (Cytiva, Marlborough, MA, USA) was used for protein purification. Eluted fractions were verified using Coomassie Blue staining and further dialyzed in PBS. Finally, the HA proteins were concentrated using the Amicon ultra filters 3kDa (Millipore), quantitated using the BCA assay, adjusted to 1 μg/μL, and stored at −80 °C.

### 2.5. Cells and Influenza Viruses 

All the cells used in this study were kept and cultured in our lab. MDCK cells were cultured in Dulbecco’s modified Eagle’s medium (DMEM; Corning, Corning, NY, USA) containing 10% fetal bovine serum (FBS; Gibco, Billings, MT, USA) and 1% 100 U/mL of penicillin-streptomycin (PS; Gibco) at 37 °C with 5% CO_2_. Sf9 cells were maintained in SF900 III insect cell medium (SFM; Gibco) supplemented with 1% PS at 27 °C.

All influenza A viruses used in this study were kindly provided by the National Influenza Center of the Chinese Center for Disease Control and Prevention (Chinese CDC). Influenza viruses were propagated in 10-day-old SPF embryonated eggs for 2 days at 35 °C and then aliquoted and frozen at −80 °C, tittering by 50% tissue culture infectious dose (TCID50).

### 2.6. Immunization and Challenges of Mice

To investigate the immunogenicity and efficacy, BALB/c mice were divided into four groups (n = 13) and intramuscularly (i.m.) immunized twice at a 21-day interval, including the H1m group, the H3m group, the commercially inactivated vaccine group, and PBS group. For the HAm immunized groups, 10 μg purified mosaic HAs was mixed evenly together with 70,000 units/mL of Interlukin-2 (IL-2) and 0.1% chitosan (Sigma-Aldrich, St. Louis, MO, USA) in 100 μL PBS. For QIV groups, 50 μL of QIV were mixed gently with 100 μL PBS. Serum was collected from these four groups at days 35-post first vaccinations. Two weeks after the second immunization, all mice were intranasally (i.n.) challenged with 10 times the mouse median lethal doses (MLD50) of H1N1 or H3N2 influenza viruses in 50 μL of PBS. Lungs of three euthanized mice from each group were collected at 4 days post-infection (dpi), tittered by TCID50. All mice were weighted and observed for clinical signs twice daily for 14 days post-challenge, while 20% body weight loss was determined to be the humane intervention point. Mice reaching this endpoint were euthanized and counted in mortality.

### 2.7. Hemagglutination Inhibition (HAI) Assay 

As previously described [28], HAI assays were used for HAI antibody tittering. Briefly, serums were diluted 1:10 with the receptor-destroying enzyme (RDE; Denka Seiken, New York, NY, USA) at 37 °C for 16 h and then inactivated at 56 °C for 30 min. Chicken or guinea pig erythrocytes were (1%) combined with 4 HA units of viruses and recorded the highest serum dilution that completely inhibited hemagglutination.

### 2.8. Microneutralization (MN) Assay 

MN assays were used to determine the neutralization antibody titers [27]. Briefly, serums were treated with RDE at 37 °C for 16 h. Serially diluted serum was incubated with 100 TCID50 influenza virus at 37 °C in 5% CO_2_ for 1 h, and then with added MDCK cells for another 18 h. All the cells were fixated with acetone and incubated successively with two antibodies: an anti-influenza virus A antibody (AB1074, Millipore) and a horseradish peroxidase-conjugated IgG (HRP-IgG) antibody (FDG007, FDbio, Hangzhou, China). TMB substrate was used to determine the microneutralization titers by reading the OD450 nm value.

### 2.9. Enzyme-Linked Immunosorbent Assay (ELISA)

ELISA assays were used to determine the IgG antibody titers [29]. Briefly, the 96-well ELISA plates (Corning) were respectively coated with 5 μg/mL of each purified inactivated virus or HAm protein overnight at 4 °C. The plates were blocked in 2% bovine serum albumin (BSA, Sigma Aldrich) in PBS with 0.05% Tween (PBST) for 60 min at RT, followed by overnight incubation with serum samples at 4 °C. Goat anti-mouse IgG, IgG1, and IgG2a antibodies (1036-05, 1071-05, and 1081-05, Southern Biotech, Birmingham, AL, USA) were then sequentially incubated for 1 h at RT and TMB substrate for 30 min, measured for the OD450 value.

### 2.10. Enzyme-Linked Immunospot (ELISpot) Assay 

ELISpot assays were used to determine the cytokines induced by splenocytes [30]. Briefly, 100 μL anti-mouse IFN-γ or IL-4 monoclonal antibody (15 μg/mL) (CT655-10 and CT657-10, U-Cytech, Utrecht, The Netherlands) was coated on ELISpot 96-well plates (Millipore) incubated at 4 °C overnight. Plates were then blocked by 1% BSA in PBS at RT for 2 h. Mouse splenocytes and inactivated A/PR/8/1934 or A/Aichi/2/1968 virus were then added and incubated at 37 °C in 5% CO_2_ for 28 h. The splenocytes were removed after the incubation, and biotin-conjugated anti-IFN-γ or anti-IL-4 antibodies (CT655-10 and CT657-10, U-Cytech) were added, followed by incubation with streptavidin-ALP at RT for 1 h. Finally, spots were detected after incubation in 5-bromo-4chloro-3-indolyl-phosphate/nitro blue tetrazolium (BCIP/NBT; U-Cytech) substrate solution for 20 min using an ELISpot Reader System (Mabtech, Stockholm, Sweden).

### 2.11. Flow Cytometry and Intracellular Cytokine Staining

To evaluate the B-cell response following vaccination, we quantified the numbers of GC B cells, plasma cells, and plasma blasts using flow cytometry [31]. Briefly, spleens were harvested and processed into single-cell suspensions. Following treatment with Fc receptor blockers, the cells were stained with fluorescently labeled antibodies including ECD-Zombie Red, PC5.5-CD3, BV421-B220, PE-FAS, APC-GL7, and PE-cy7-CD138. Subsequently, the stained cells were collected by CytoFLEX S Flow Cytometer (Beckman Coulter, Brea, CA, USA).

Cytokines expression was evaluated using flow cytometry for T cells in single-cell suspensions from spleen as previously described [31]. Splenocytes were stimulated in medium containing 10 μg/mL purified A/Brisbane/02/2018, A/Hong Kong/4801/2014, A/PR/8/1934, or A/Aichi/2/1968 virus for 2 h at 37 °C, respectively, and then incubated with BFA for another 14 h. The cells were stained with murine antibodies for phenotype (PC5.5-CD3, BV421-CD4, and FITC-CD8) and cytokine expression (PE-IFN-γ, BV605-IL-2, BV650-IL-4, and PE-cy7-TNF-α), along with a dead or live cell dye (ECD-Zombie Red, Biolegend, BD, San Diego, CA, USA). Finally, the cells were collected by CytoFLEX S Flow Cytometer, and the data were processed with CyExpert 2.4 (Beckman Coulter) and FlowJo V10.8 (Tree Star).

### 2.12. Histological Examination of the Mice’s Lung

Lung tissues from both infected and uninfected mice were subjected to formalin fixation, paraffin embedding, and sectioning, followed by staining with hematoxylin and eosin.

### 2.13. Statistical Analysis

Data analysis was performed using GraphPad Prism 8.0.2 software. Statistical significance was assessed using one- or two-way ANOVA followed by multiple comparison tests. Data were presented as the mean with standard error of the mean (SEM). A *p*-value < 0.05 was considered statistically significant.

## 3. Results

### 3.1. Mosaic HA Sequences Construction and Structural Prediction

**Figure 1:**
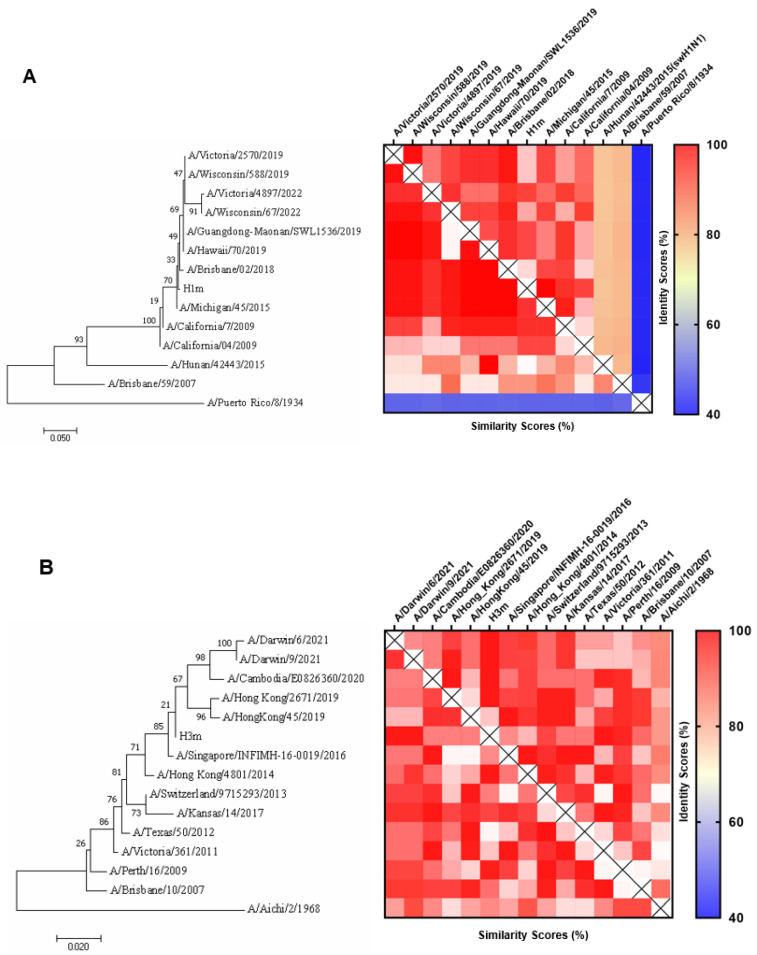
Phylogenetic relationship (left), percent identity, and percent similarity (right) of the representative influenza strains hemagglutinin (HA) proteins and mosaic recombinant HA antigens. (**A**) H1m (**B**) H3m. All were aligned using ClustalW alignment, and a maximum-likelihood phylogenetic tree was constructed using MEGA7.

**Figure 2:**
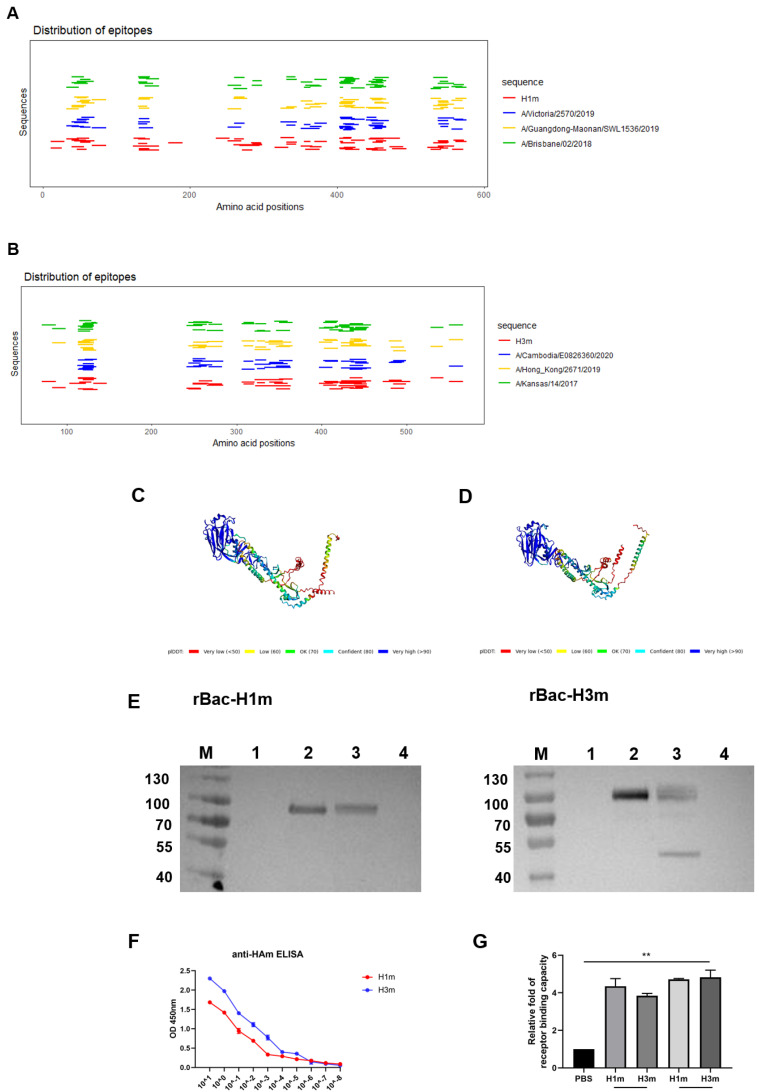
Generation and characterization of recombinant mosaic HA proteins. (**A**,**B**) The distribution of epitopes in mosaic and influenza vaccine strain sequences. The lines indicate known T-cell epitopes that were found in those sequences by use of the Immune Epitope Database (IEDB). (**C**,**D**) The three-dimensional (3D) structure model of monomer to Mosaic HA antigen. The results indicated a 3D structure model of monomer to (**C**) H1m (**D**) H3m. Mosaic recombinant antigen sequences were submitted to the AlphaFold2 server to construct the homology-derived conformational model. (**E**) Recombinant baculovirus (rBVs) rBac-H1m and rBac-H3mwere successfully identified using Western Blot (WB) by HA-specific antibodies in Sf9 cells. (lane M: marker, lane1: P1 baculovirus stock supernatant, lane2: P2 baculovirus stock supernatant, lane3: P2 baculovirus stock cell lysate, lane4: negative control). (**F**) ELISA using CR9114, a monoclonal antibody (mAb) that recognizes conformational epitopes on the HA stalk domain. (**G**) Recombinant mosaic HA proteins can bind to both SAα-2, 3Gal and SAα-2, 6Gal sialic acid receptors. Data are presented as the mean with standard error (SEM), ** *p* < 0.01.

We obtained two mosaic HA amino acid sequences of H1N1 and H3N2 named HAm (H1m and H3m), as described previously, via the genetic algorithm of the mosaic vaccine design strategies [24]. Instead of setting the epitope length to “12” to obtain mosaic sequences covering more CD4+ Th cell epitopes, we aligned them with the vaccine strain sequences of the last decade. Phylogenetic analysis revealed that H1m and H3m were close to the annual influenza vaccine strains, indicating their potential as vaccine antigens (Figure 1). When aligned with vaccine strain sequences of the last decade, H1m and H3m differed between four to twenty-four and three to sixteen amino acids, respectively. A/Michigan/45/2015 and A/Singapore/INFIMH-16-0019/2016 HA proteins were most similar to H1m and H3m, while A/Victoria/4897/2022, A/Darwin/6/2021, and A/Darwin/9/2021 HA proteins were less similar (Figures S1 and S2). We also presented the distribution of T-cell antigenic epitopes within the mosaic HA and vaccine strain HA sequences (Figure 2A,B). To evaluate whether designed sequences could be folded into stable structures correctly, we simulated the three-dimensional (3D) model of recombinant monomeric mosaic antigen using AlphaFold2. (Figure 2C,D).

To generate the recombinant mosaic HA proteins, we modified the protein as previously described [20,23]. Briefly, we replaced the original HA signal peptide with a gp67 signal peptide at the N-terminus for efficient expression and secretion in Sf9 cells. Additionally, we modified the C-terminal sequences by replacing the original intracellular domain with a sequential thrombin cleavage motif, GCN4 adaptor, and octa-histidine tag to enhance trimer formation and purification (Figures S1 and S2). The HAm proteins were expressed using the Bac-to-Bac baculovirus expression system; Coomassie Blue staining and Western blotting confirmed the successful expression of H1m and H3m (Figure 2E and Figure S3). When purified, we verified that the H1m and H3m proteins were recognized by CR9114, a monoclonal antibody (mAb) that binds conformation-sensitive epitopes on the HA stalk (Figure 2F). Furthermore, we examined the binding capacity of HAm proteins to sialic acid receptors, showing that HAm can bind to both SAα-2, 3Gal and SAα-2, 6Gal sialic acid receptors, with stronger bindings to SAα-2, 6Gal sialic acid receptors (Figure 2G).

### 3.2. Recombinant Mosaic HAs Enhanced HAI Breadth and Humoral Immune Responses Compared with That Induced by QIV Vaccines in Mice

**Figure 3:**
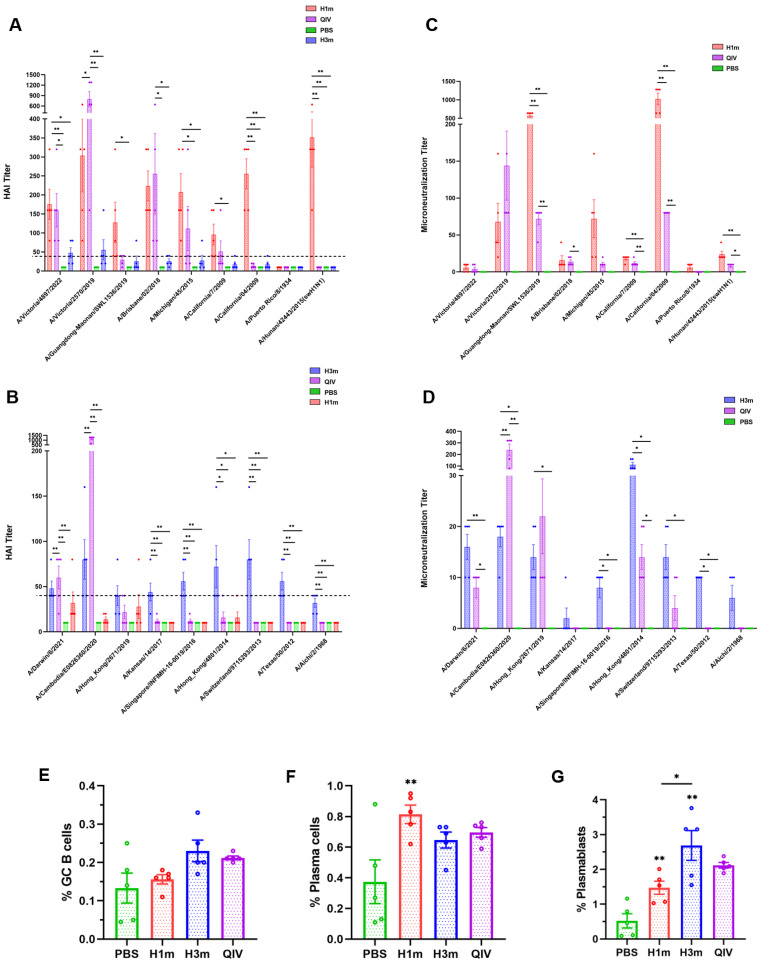
Recombinant mosaic HA influenza vaccine immunization elicits cross-reactive antibody responses. Recombinant mosaic HA-based vaccine was administered to BALB/c mice and compared to those vaccinated with QIV. (**A**,**B**) The HAI assay was used to assess the antibody response against H1N1 and H3N2 representative strains in mice following boost immunization. (**C**,**D**) The microneutralization (MN) assay measured the neutralizing antibody response to H1N1 and H3N2 representative strains in mice after boost immunization. The B-cell response was assessed by flow cytometry detection of (**E**) GC B cells, (**F**) plasma cells, and (**G**) plasma blasts. Data are presented as the mean with SEM (n = 5; one-way ANOVA with Tukey multiple comparison), * *p* < 0.05, ** *p* < 0.01.

**Figure 4:**
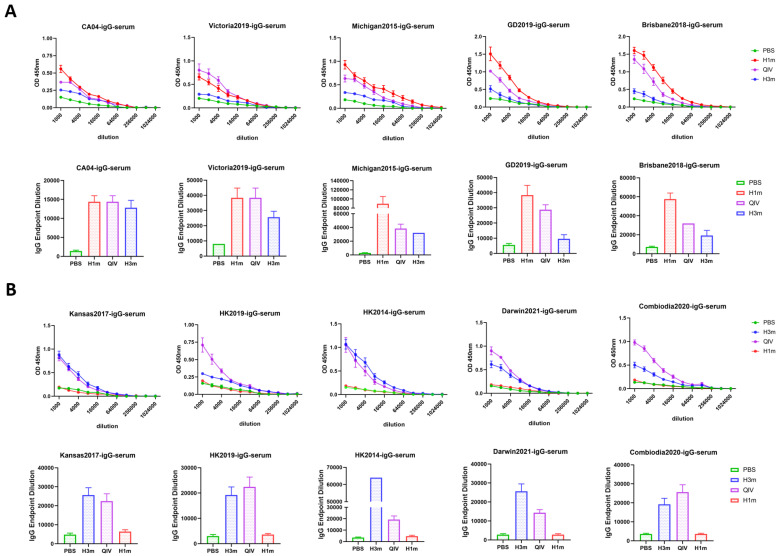
Recombinant mosaic HA influenza vaccine immunization elicits higher antibody titers in mice against H1 and H3 representative viruses. Antibody binding was determined by ELISA assay. (**A**) ELISA against H1 representative strains A/California/04/2009 (CA04), A/Victoria/2570/2019 (Victoria2019), A/Michigan/45/2015 (Michigan2015), A/Guangdong-Maonan/SWL1536/2019 (GD2019), and A/Brisbane/02/2018 (Brisbane2018). (**B**) ELISA against H3 representative strains A/Kansas/14/2017 (Kansas2017), A/Hong Kong/45/2019 (HK2019), A/Hong Kong/4801/2014 (HK2014), A/Darwin/9/2021 (Darwin2021), and A/Cambodia/E0826360/2020 (Cambodia2020). Data are presented as the mean with SEM.

In order to determine the breadth of antibodies elicited by the HAm antigens, 6-week-old female BALB/c mice were administered a prime-boost schedule with HAm, QIV, or PBS over a span of 21 days, respectively. Serum was collected on day 35 and tested for hemagglutination inhibition (HAI) antibody against both representative ancestral strains and seasonal H1N1 and H3N2 subtype vaccine strains (2009–2024) (Figure 3A,B). The results showed that immunization with both H1m and H3m effectively induced potent cross-reactive antibodies that blocked the virus binding to the turkey or guinea pig red blood cells, except for the HAI titer of serum that remained < 40 HAI units to A/Puerto Rico/8/34 (PR8) and A/Aichi/1968 (X-31) (in humans, an HAI titer ≥ 40 is often used as a threshold protective titer). Mice immunized with the HAm vaccine exhibited HAI titers ranging from 40 to 640 units against 16 out of 18 tested strains. This observation underscores the ability of the HAm to elicit robust cross-reactive immunity against a subset of mismatched strains, with particular efficacy against A/Hunan/42443/2015, a swine influenza virus exhibiting an HAI titer of ≤640. Conversely, mice vaccinated with the QIV demonstrated limited cross-reactivity with mismatched viruses, as depicted in Figure 3. Specific IgG antibodies produced in the serum of vaccinated mice exhibited similar results (Figure 4). This finding underscores the superior cross-reactive immunity induced by the HAm. Further investigation involved the measurement of neutralizing antibody titers in vaccinated mice using the microneutralization (MN) assay. Figure 3 illustrates that mice vaccinated with HAm exhibited high levels of neutralizing antibody titers following two shots of immunization. These neutralizing antibodies effectively curtailed the replication of multiple virus strains when compared to mice immunized with QIV and PBS, resulting in fold-increases in nAb titers (GMTs) ranging from 1.14 to 14.40 for select strains (Figure 3C,D). This result suggests that serum obtained from HAm-vaccinated mice possesses noteworthy homologous neutralizing activity, with a potential protective mechanism linked to host cell membrane fusion. We further detected whether HAm produces humoral immune responses to heterologous influenza virus, and found that H3m-vaccinated mice produced HAI antibody titers >40 to some H1N1 viruses, and that mice immunized with H1m also elicited cross-reactive HAI antibody to some H3N2 viruses (Figure 3A,B). The ELISA results also showed that mice immunized with H3m elicited cross-reactive IgG antibodies to the heterologous influenza virus (Figure 4). In conclusion, these results support the hypothesis that the HAm vaccine formulation confers broad cross-reactivity potential. Flow cytometric analysis of changes in splenic B cells after immunization of mice also proves the above point. The gating strategies are shown in Figure S4. The increase in the number of germinal center (GC) B cells, plasma cells, and plasma blasts following HAm immunization in mice improves both the quality and efficiency of humoral immune responses (Figure 3E–G).

### 3.3. Recombinant Mosaic HA Proteins Elicited More T Cell Immune Responses Compared with That Induced by QIV Vaccines in Mice

**Figure 5:**
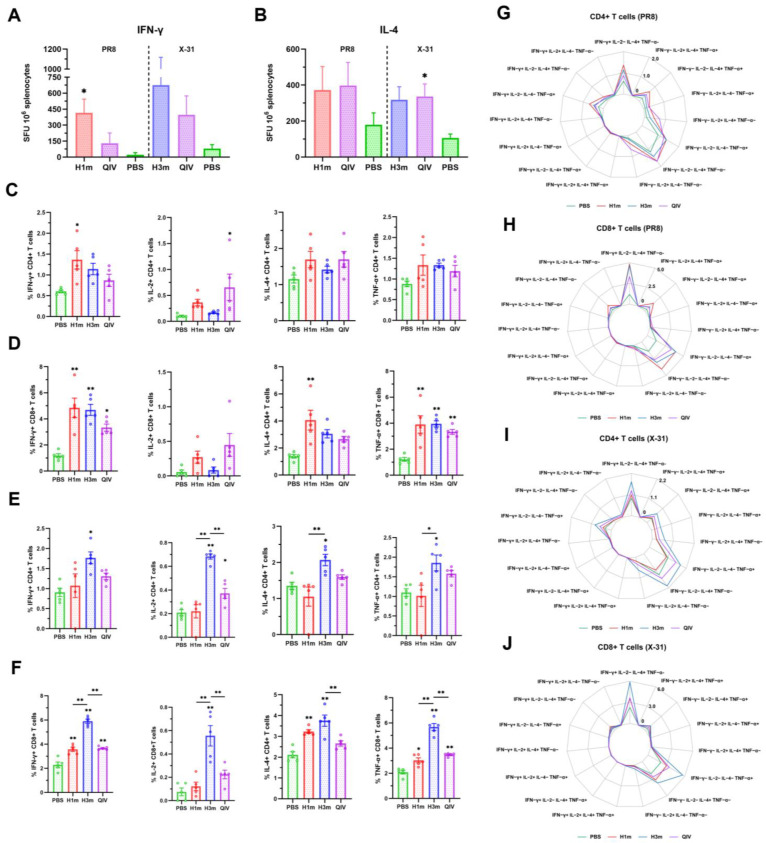
Recombinant mosaic HAs influenza vaccine immunization elicits T cell responses. BALB/c mice were vaccinated with Recombinant mosaic HA-based vaccine and compared to QIV-vaccinated mice. (**A**,**B**) Recombinant mosaic HA-based vaccine elicits T-cell responses in mice. Splenocytes restimulated with inactive A/Puerto Rico/8/34 (PR8) and A/Aichi/2/1968 (X-31) virus were analyzed for cellular immunity using an IFN-γ (**A**) and IL-4 (**B**) ELISpot assays (n = 5). (**C**–**F**) Intracellular cytokine staining of splenocytes for (**C**,**E**) IFN-γ+, IL-2+, IL-4+, or TNF-α+ CD4+ T cells and (**D**,**F**) IFN-γ+, IL-2 +, IL-4+, or TNF-α+ CD8+ T cells following (**C**,**D**) A/Brisbane/02/2018 (Brisbane2018) or (**E**,**F**) A/Hong Kong/4801/2014 (HK2014) stimulation. Data are presented as the mean with SEM (n = 5; one-way ANOVA with Tukey multiple comparison), * *p* < 0.05, ** *p* < 0.01. (**G**–**J**) Radar plot shows polyfunctionality of the CD4+ or CD8+ T cell response. Geometric mean frequencies are displayed.

T-cell response is essential in combating both homologous and heterologous influenza viruses. To further understand the immune response mechanism underlying the protective effect of HAm, we investigated the function of T cells during this process. We performed ELISpot assays to measure IL-4 or IFN-γ-secreting cells and found that HAm immunization significantly increased the number of IFN-γ-producing splenocytes when compared to both QIV and PBS groups. Furthermore, both the HAm and QIV groups induced high levels of IL-4-producing splenocytes with no significant differences (Figure 5A,B). These findings suggest that immunization with HAm can activate T helper 1 (Th1) and T helper 2 (Th2) cells, which are necessary for adaptive immune responses.

Thirty-five days post-immunization, flow cytometry with ICS was performed on splenocytes from immunized mice, which were stimulated with purified viruses. Representative flow cytometry images showing CD8+ IFN-γ+, IL-2+, IL-4+, TNF-α+, and CD4+ IFN-γ+, IL-2+, IL-4+, TNF-α+ T cells under A/Brisbane/02/2018 virus stimulation are displayed in Figure S5. Gating strategies are shown in Figures S6 and S7. Robust T cell responses post-immunization with all mosaic HAs were observed. The CD4+ T cell response to the purified viruses showed a Th1 phenotype, with significantly higher frequencies of CD4+ T cells expressing IFN-γ (5.584%, 3.550%) and TNF-α (3.910%, 3.510%) in HAm-immunized mice compared to the QIV group (Figure 5C,E). CD8+ T cell responses were more pronounced, with higher frequencies of IFN-γ (7.824%, 6.398%) and TNF-α (7.352%, 7.312%) secreting cells in the HAm group compared to QIV (Figure 5D,F). Surprisingly, splenocytes from mice immunized with H1m triggered cross-T-cell immunity against purified A/Hong Kong/4801/2014 virus, whereas splenocytes from mice immunized with H3m triggered cross-T-cell immunity against purified A/Brisbane/02/2018 virus, which is important for guiding vaccine design (Figure 5C–F). The antigen-specific T cell responses were characterized by their polyfunctionality. CD4+ T cells predominantly displayed IFN-γ+ IL-2-IL-4-TNF-α+, IFN-γ+ IL-2-IL-4+ TNF-α-, and IFN-γ-IL-2-IL-4-TNF-α+ phenotypes (Figure 5G,I). And the dominant CD8+ T cell population was also highly polyfunctional, with phenotypes of IFN-γ+ IL-2-IL-4+ TNF-a-, IFN-γ-IL-2-IL-4-TNF-a+, and IFN-γ-IL-2-IL-4+ TNF-a- (Figure 5H,J). Additionally, when mouse splenocytes were stimulated with A/Puerto Rico/8/34 (PR8) and A/Aichi/2/1968 (X-31) purified viruses, similar T-cell responses were observed (Figure S8). 

### 3.4. Recombinant Mosaic HA Proteins Immunized Mice Were Protected from Lethal Virus Challenges

**Figure 6:**
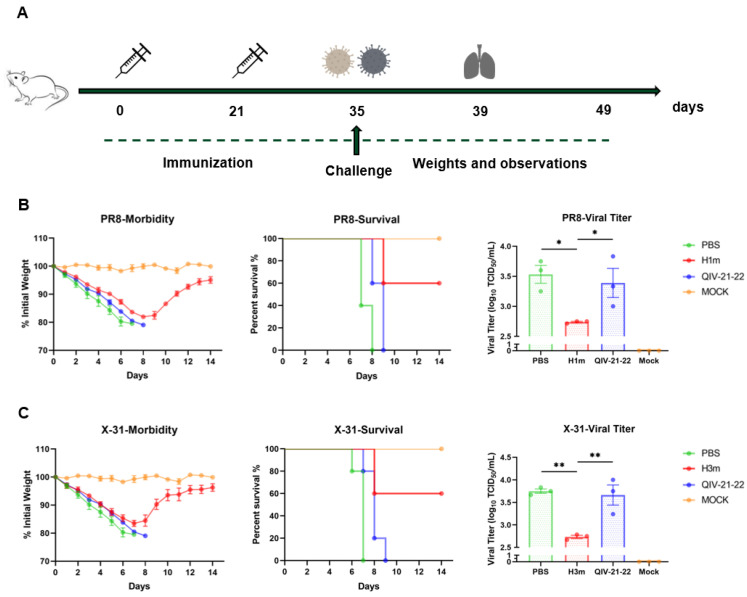
The prophylactic activity of recombinant mosaic HAs influenza vaccine in an influenza A virus challenge model. (**A**) Diagram showed experimental plan. Mice received a boost immunization with recombinant mosaic HA proteins or quadrivalent inactivated influenza vaccine (QIV) or PBS and were infected with A/Puerto Rico/8/34 (PR8) or A/Aichi/2/1968 (X-31) intranasally. Three mice per group were euthanized at 4 days post-infection (dpi). Lungs were harvested to determine viral titer and histopathology. (**B**,**C**) Graph showed body weight loss and survival rate of mice (n = 5) and mean lung virus titer (n = 3) at 4 dpi. Data are presented as the mean with SEM. * *p* < 0.05, ** *p* < 0.01.

To evaluate the antiviral effect of HAm, 6-week-old female BALB/c mice were immunized twice at a 21-day interval with HAm, QIV, or PBS via intramuscularly (i.m.). The mice were then challenged with lethal doses of 10 MLD50 (50% mouse lethal dose) mouse-adapted viruses of A/Puerto Rico/8/34 (PR8; H1N1) and A/Aichi/1968 (X-31; H3N2) via intranasal (i.n.) inoculation (Figure 6A), respectively. The efficacy of vaccine protection was evaluated for 14 days by recording the body weight loss and survival rate. 

For the PR8 challenge group, 60% of the H1m immunized mice exhibited complete protection and only experienced mild weight loss, while the QIV group succumbed to infection after 9 days (0% survival). Similar to the results obtained in the PR8 challenge, for the X-31 challenge group, weight loss was minimal in the H3m group, whereas the QIV vaccine group had 0% survival. Taken together, we found that the HAm vaccine provided better protection to homologous ancestral strains (Figure 6B,C). Additionally, virus clearance was assessed in another group of vaccinated mice at 4 dpi. The HAm-immunized mice had significantly reduced lung virus titers compared to the QIV and PBS groups in the case of the lethal dose influenza virus challenge (Figure 6B,C). Furthermore, histological analysis of lung tissue showed apparent alveolar destruction, connective tissue hyperplasia, inflammatory cell infiltration, and alveolar wall thickening in the QIV and PBS groups, while the lungs of mice vaccinated with HAm only showed mild pathogenic changes like lighter cellular infiltration and inflammation (Figure S9). While viral titers in the lungs do not always correlate with morbidity [32], the strong inverse correlations between viral titers and pulmonary inflammatory cytokines and infiltrates suggest that the HAm played an essential role in reducing the severity of infection. Overall, these data demonstrated that HAm could provide significant protection against lethal influenza virus challenges, suggesting that immunization with HAm effectively protects against lethal influenza virus challenges and reduces the damage caused by influenza virus infection in the lung tissues. This represents an advance on conventional QIV vaccines, which fail to protect from vaccine-mismatched viruses with antigenic drift.

## 4. Discussion

Vaccination is widely recognized as the most effective method to prevent morbidity and mortality caused by influenza. However, the effectiveness of current influenza vaccines is variable, and seasonal influenza remains a significant public health issue worldwide. Antigenic drift, primarily resulting from mutations in HA protein, is the primary cause of seasonal influenza vaccine mismatches and failures [33,34]. In this study, we aimed to develop a universal influenza vaccine using recombinant mosaic HA proteins to provide broader protection than currently available seasonal influenza vaccines. 

Antibodies that can mediate HAI are known to be effective in providing protection against influenza and are widely recognized as good predictors of vaccine efficacy [35]. Therefore, the ability to elicit high levels of HAI antibodies is considered a crucial factor in new vaccine licensing in numerous countries [36,37]. In this study, we used H1N1 and H3N2 HA sequences from 2009 to 2021 to generate mosaic HA antigens. We selected 18 strains that represent the typical human-infection spectrum of IAV in challenging situations. Following two doses of immunizations, the HAm elicited cross-reactive HAI antibodies against 17 out of 18 selected homologous, heterologous, or heterosubtypic IAV strains, while the QIV vaccine showed limited cross-reactivity. Moreover, the mosaic HAs vaccine induced significantly higher HAI antibody titers than the QIV vaccine, which may offer more comprehensive protection in high-risk populations, especially in pediatric or elderly populations, as previously reported [38,39]. Thus, we believe that the HAm antigens have broad spectrum potential. 

Antibodies targeting heterosubtypic antigens can help suppress interspecies transmission and prevent zoonotic influenza virus infection in humans. In this study, the HAm induced extremely high HAI titers against A/Hunan/42443/2015 (H1N1), a swine influenza virus strain, even surpassing the titers against other seasonal vaccine strains. This suggests that the mosaic antigens may cover conserved epitopes of some heterosubtypic strains, indicating potential for cross-host protection and transmission. However, despite administering the same dose of both antigens, the H3N2 subtype produced a lower antibody response magnitude than the H1N1 subtype (Figure 3). The frequent genetic and antigenic changes in H3N2 may contribute to poorly conserved antigenic epitopes, with some epitopes losing functionality due to mutations [40]. Increasing the dose of poorly immunogenic HA subtypes has been shown to overcome immune response limitations in polyvalent vaccine formulations [41,42]. Therefore, optimizing the ratio of H1 and H3 antigen doses to balance the immune response levels in vaccine recipients is a goal for future vaccine development. 

In this study, the HAm vaccines elicit both humoral and cellular immunity. Consistent with previous studies [43], vaccinated mice serum samples collected in this study also showed a significant correlation between HAI and MN titers. Specifically, for certain viral strains, vaccination with the HAm demonstrated the induction of elevated levels of both HAI and MN antibodies. This dual effect effectively hindered the virus from attaching to host cells, thereby neutralizing the virus and preventing pathogenic infection. These findings strongly suggest that HAm vaccination plays a pivotal role in mitigating the occurrence and dissemination of pathogenic infections. Although currently commercial influenza vaccines mainly focus on humoral responses, T-cell-mediated immunity is required to clear most viral infections and maintain long-term immunity [44,45,46]. Antigen-specific T cells undoubtedly play a key role in the protection and produce more extensive cross-reactivity responses than antibodies [47]. In our study on vaccinated mouse serums, we found that HAm induced the high frequency of T-cell responses to historical virulent strains, promoting T-lymphocyte proliferation and enhancing the function of CD4+ and CD8+ T cells, which is one of the sufficient reasons why HAm provided robust protection against lethal challenge. We observed that HAm immunization triggered cross-reactivity of T cells across subtypes in mice. Cytokines produced by CD8+ and CD4+ T cells were dominated by either IFN-γ or IL-4, with the antiviral role of IFN-γ being well reported, but IL-4 produced by T cells also promotes a more rapid neutralizing antibody response to heterologous infections [48]. This suggests that multiple components of immunity may act synergistically to limit viral pathogenesis. 

It is noteworthy that our calculated mosaic HA sequence is designed to encompass the broadest array of CD4+ T cell epitopes. However, immunizing mice with the mosaic HA antigen not only elicited the production of IL-4 but also led to a substantial release of IFN-γ. This intriguing discovery suggests the activation of both CD4+ and CD8+ T cell subsets upon immunization with HAm, indicating a simultaneous engagement of both T-helper (Th) and cytotoxic T lymphocyte (CTL) pathways within the host’s immune response. CD4+ T cells can differentiate into Th1 and Th2 cells, which have critical roles in the immune response, including clearance of viral infections and regulating immunoglobulin class switching [49,50]. CD8+ T cells enhance antiviral responses by destroying virus-infected cells through the CTLs [46,51]. It has long been recognized that influenza virus-specific CD8+ T cells can cross-react with various subtypes of influenza A virus [52,53]. This implies that CD8+ T cells can be induced by vaccination to provide heterosubtypic immunity. In addition, influenza virus-specific CD4+ T cells have been shown to have an essential role in protection against infection by indirectly supporting B-cell affinity maturation, eliciting CD8+ T-cell responses, and protecting in the absence of neutralizing antibodies in a mouse model [53,54,55]. Our results showed that HAm could significantly enhance the Th1 and Th2 cell immune response by increasing the differentiation of IFN-γ and IL-4-producing cells and the expression of antigen-specific cytokines. Moreover, Th1 cells promoted the differentiation of antigen-specific CD8+ T cells into CTL effector cells and killed virus-infected cells specifically. Limited by the murine-adapted strain, we used only one homologous strain in the challenge experiments, which caused the need for more data on protection in homologous or heterologous strains. In the following study, we will evaluate the effect of HAm on homologous and heterologous strain protection. 

One limitation of our study design is that the durability of the vaccine response was not evaluated. This will be important to determine the frequency of vaccination required to provide long-term protection. Additionally, we acknowledge that the use of five mice per group may limit the statistical power of the study, particularly in detecting subtle effects. This represents a limitation in the robustness of our findings, and future studies should include larger sample sizes and independent replications to validate the results and ensure generalizability. This candidate vaccine is currently undergoing further development in the preclinical stage, with the potential for large-scale production. It is anticipated that this vaccine may induce a more robust and enduring immune response when compared to a commercially inactivated influenza vaccine. The adjuvants employed in our study have also undergone comprehensive preclinical investigations before clinical application. In summary, soluble expression of HAm in the Bac-to-Bac system indicated that it could be applied in rapid and large-scale vaccine production. Our evaluation efforts showed that this HAm vaccine design preserved the immunogenicity of the antigens and elicited strong protective efficacy primarily via HAI antibodies, neutralizing antibodies, and T cell immune responses. More importantly, the highly cross-reactive antibody and T-cell responses of HAm showed that it could become a universal vaccine candidate with broad protection. Further, the strategy used in this study to generate the HAm is also available for the production of recombinant IBV HA or NA; ideally, a future HA-based quadrivalent vaccine will contain H1, H3, and IBV HA to provide optimal protection.

## 5. Patents

Some data in this manuscript will be included in the patent application (CN115894636A).

## Data Availability

The Mosaic vaccine designer algorithm used in this study is freely available at https://www.hiv.lanl.gov/content/sequence/MOSAIC/makeVaccine.html (accessed on 1 December 2023). All sequences used to create the Mosaic immunogens are freely available through the Influenza Virus Database at https://www.ncbi.nlm.nih.gov and https://gisaid.org (accessed on 1 December 2023). All data are available in the main text or the Supplementary Materials. All other relevant data will be provided by the corresponding author upon request.

## Supplementary Materials

The following supporting information can be downloaded at: https://www.mdpi.com/article/10.3390/vaccines12091008/s1, Figure S1: Sequences and structural analysis of H1m and influenza vaccine strains; Figure S2: Sequences and structural analysis of H1m and influenza vaccine strains; Figure S3: Generation and characterization of recombinant mosaic HA proteins; Figure S4: Flow cytometric gating strategies of expressing the GC B cells, plasma cells, and plasma blasts; Figure S5: Representative flow images of CD4+ and CD8+ T cells. Figure S6: Flow cytometric gating strategies of expressing cytokines of CD4+ T cells; Figure S7: Flow cytometric gating strategies of expressing cytokines of CD8+ T cells; Figure S8: Recombinant mosaic HAs influenza vaccine immunization elicits T cell responses. Figure S9: Lung histology of challenged mice; Supplementary Data S1: The HA Mosaic Sequences.
