# Supplementary material for: A Recombinant Mosaic HAs Influenza Vaccine Elicits Broad-Spectrum Immune Response and Protection of Influenza a Viruses
Source: Vaccines (Basel). 2024 Sep 2;12(9):1008. doi: 10.3390/vaccines12091008 (PMC11435869; doi:10.3390/vaccines12091008)
Supplement: Supplementary file 1 [file vaccines-12-01008-s001.zip › vaccines-3078844-supplementary.pdf]

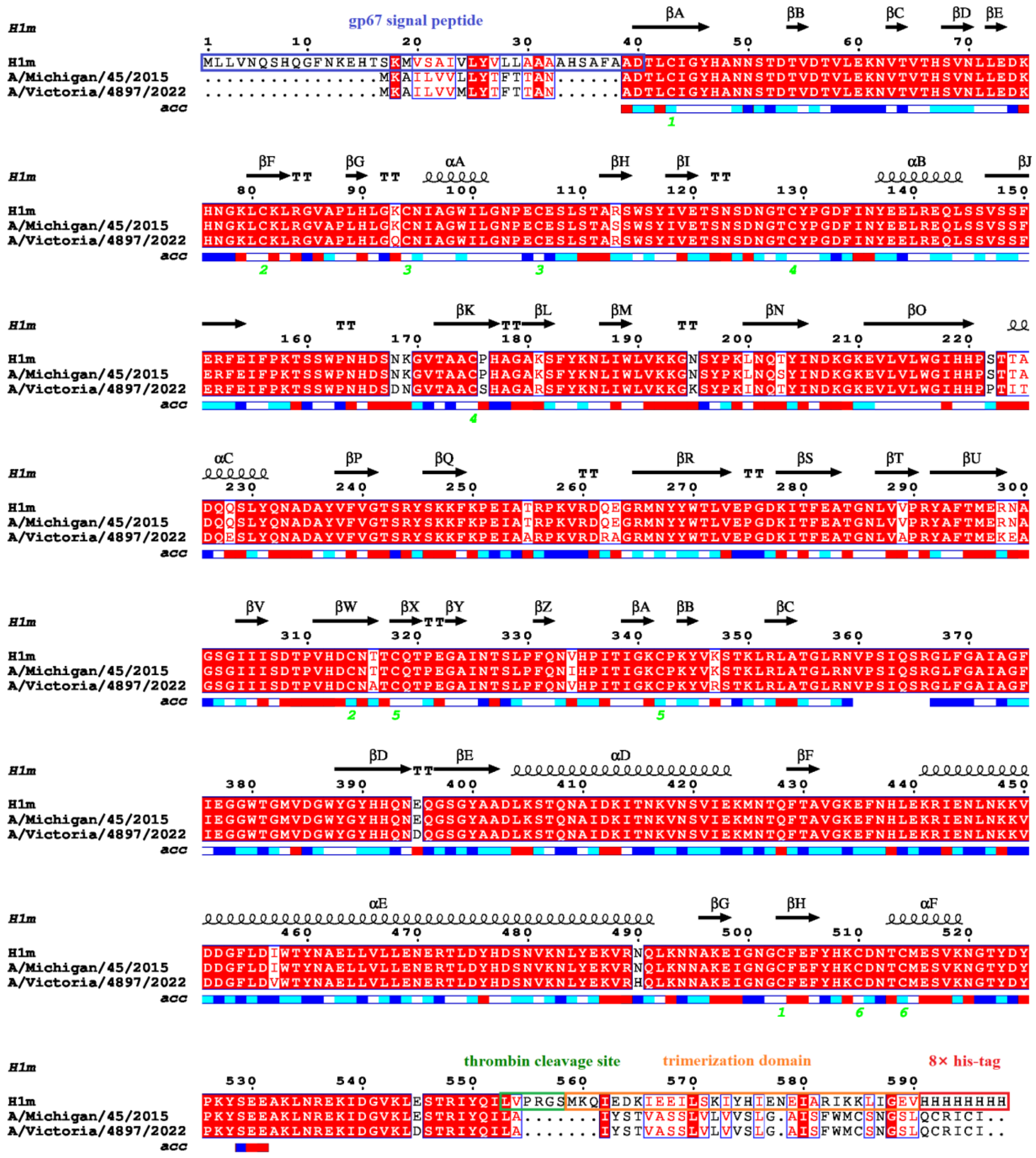

Figure S1. Sequences and structural analysis of H1m and influenza vaccine strains. Sequence alignment between H1m, A/Michigan/45/2015 and A/Victoria/4897/2022. The graph displays labeled elements, including the gp67 signal peptide, the thrombin cleavage site, the trimerization domain, and the octa-histidine purification tag. Identical amino acids are highlighted to denote their similarity.

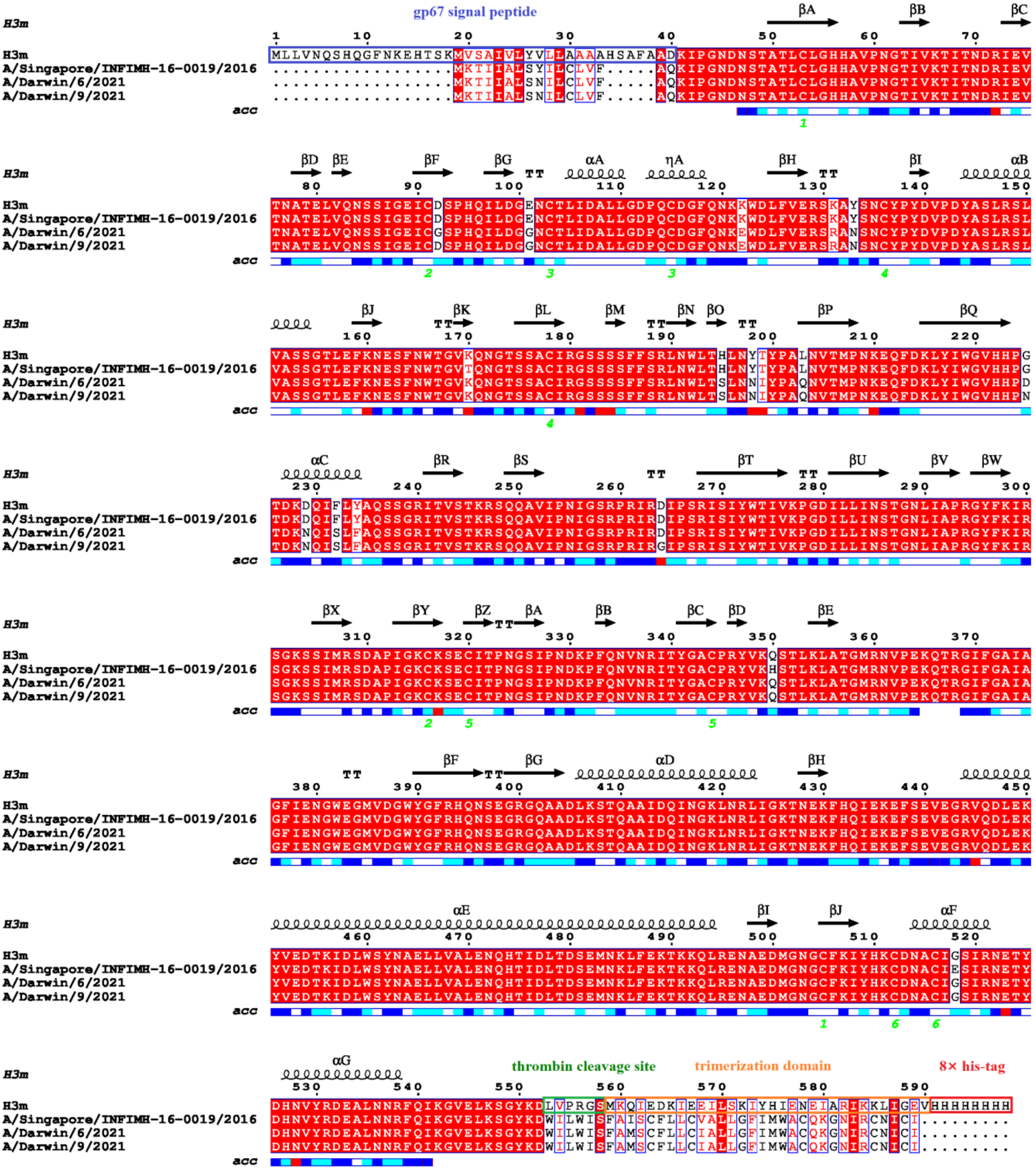

Figure S2. Sequences and structural analysis of H1m and influenza vaccine strains. Sequence alignment between H3m, A/Singapore/INFIMH-16-0019/2016, A/Darwin/6/2021 and A/Darwin/9/2021. The graph displays labeled elements, including the gp67 signal peptide, the thrombin cleavage site, the trimerization domain, and the octa-histidine purification tag. Identical amino acids are highlighted to denote their similarity.

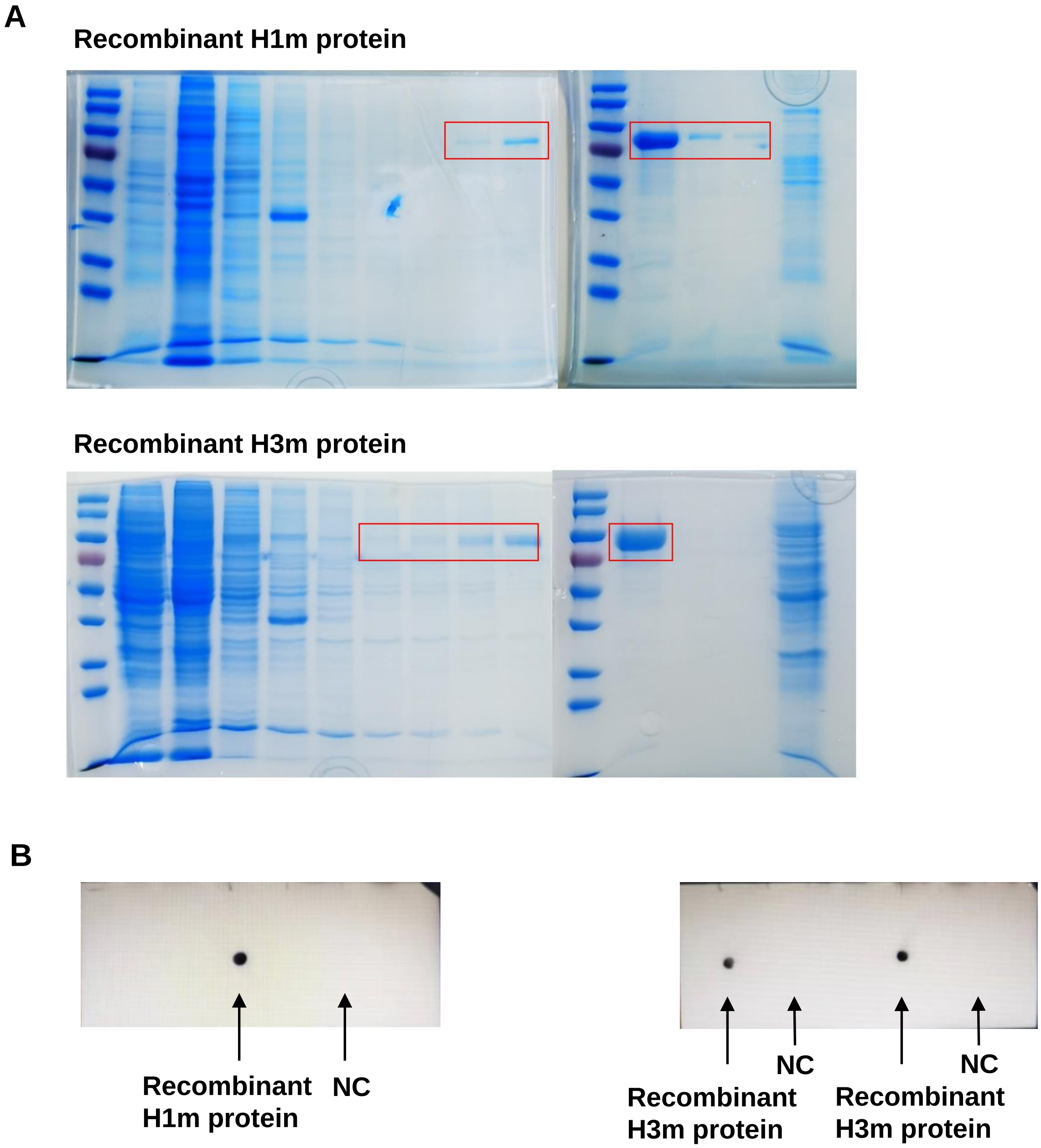

Figure S3. Generation and characterization of Recombinant mosaic HA proteins. (A) SDS-PAGE under reducing conditions. Eluted fractions containing H1m and H3m proteins were verified using Komas Brilliant Blue staining. H1m and H3m show monomeric structures at 70-100 kDa. (B) Verification of correct expression of recombinant mosaic HA proteins using protein spot hybridization.

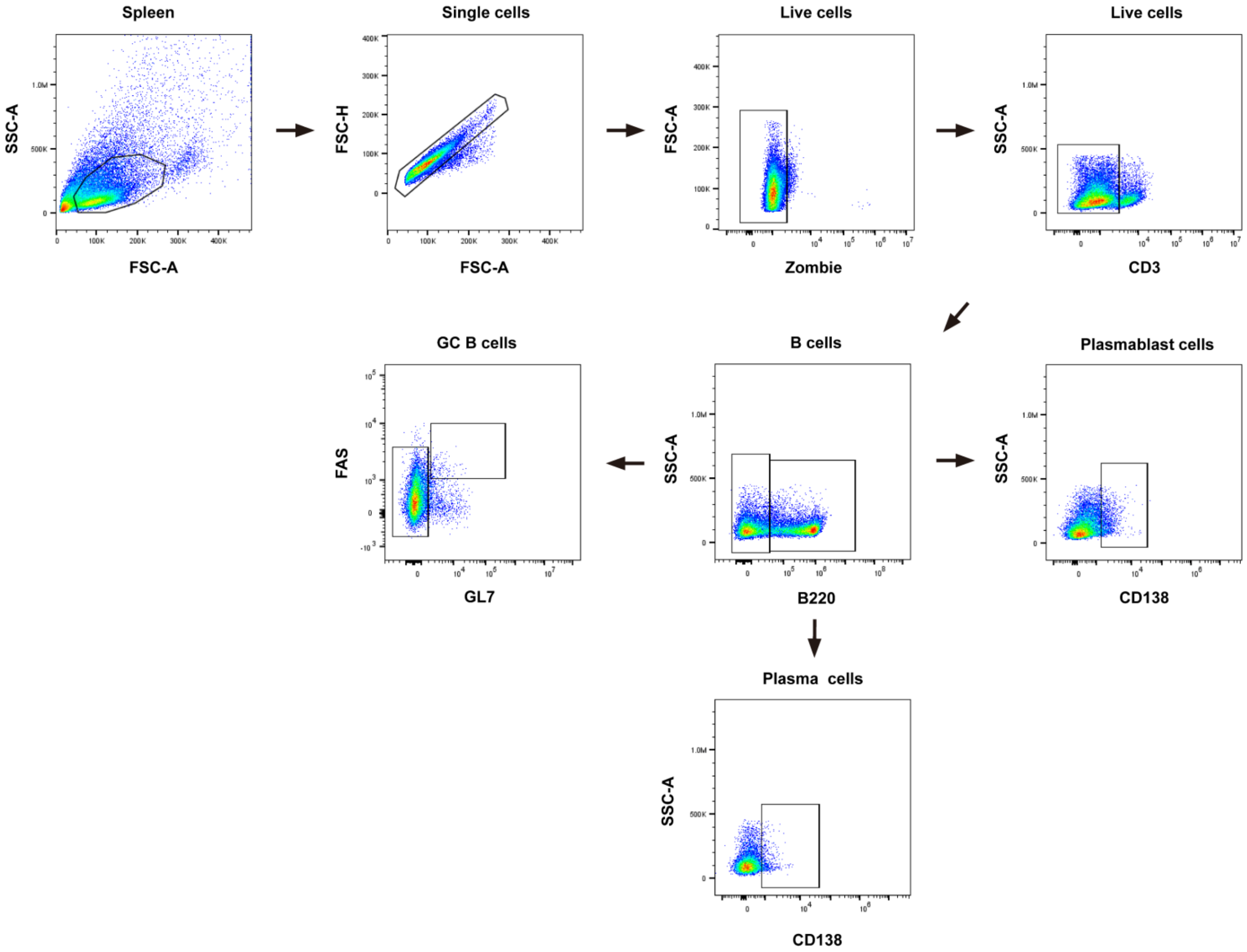

Figure S4. Flow cytometric gating strategies of expressing the GC B cells, plasma cells, and plasmablasts.

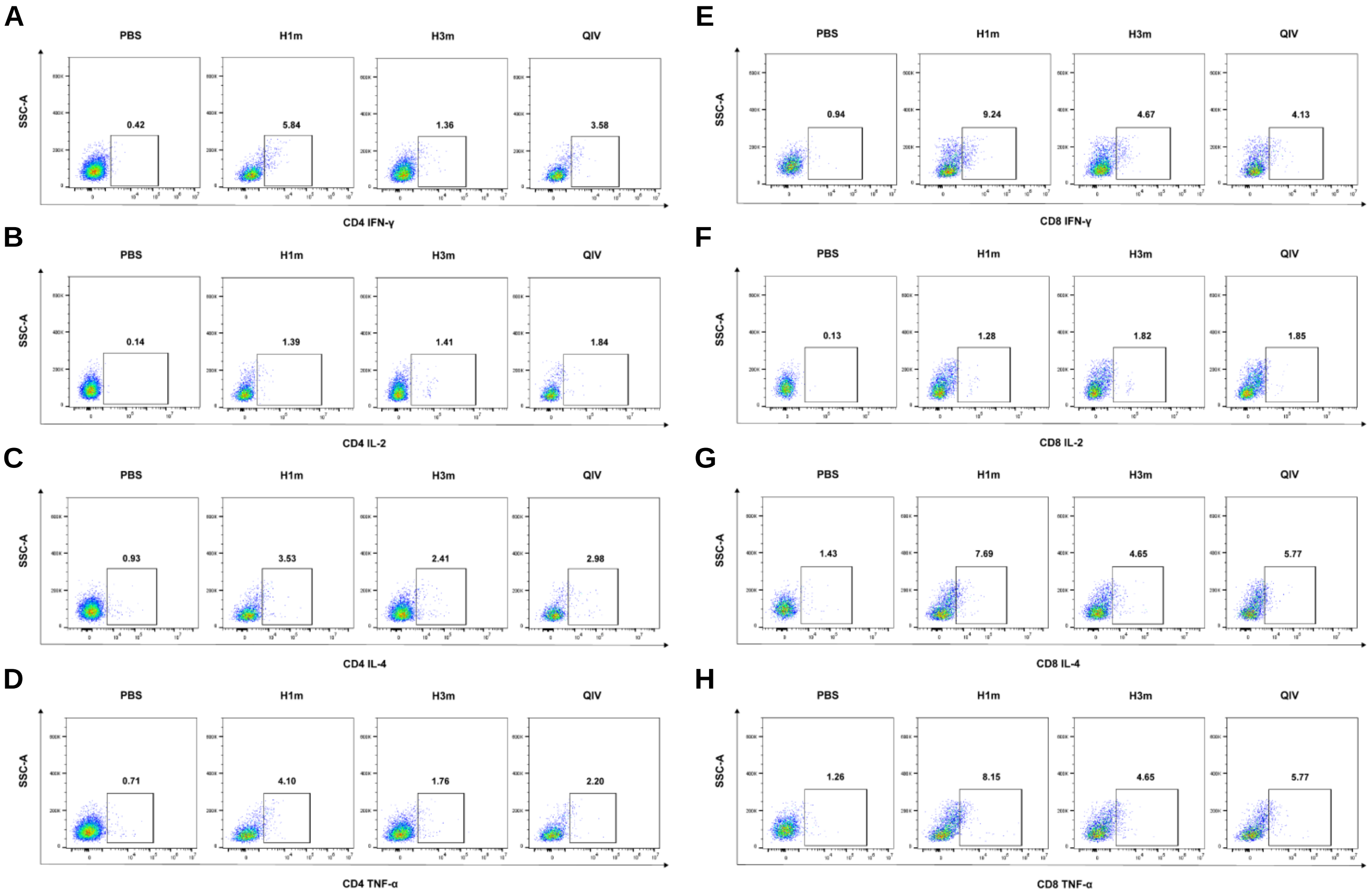

Figure S5. Representative flow images of CD4+ and CD8+ T cells. (A) CD4+ IFN-γ+ T cells, (B) CD4+ IL-2+ T cells, (C) CD4+ IL-4+ T cells, (D) CD4+ TNF-α+ T cells, (E) CD8+ IFN-γ+ T cells, (F) CD8+ IL-2+ T cells, (G) CD8+ IL-2+ T cells, and (H) CD8+ IL-2+ T cells generated by virus A/Brisbane/02/2018 (Brisbane2018) stimulation.

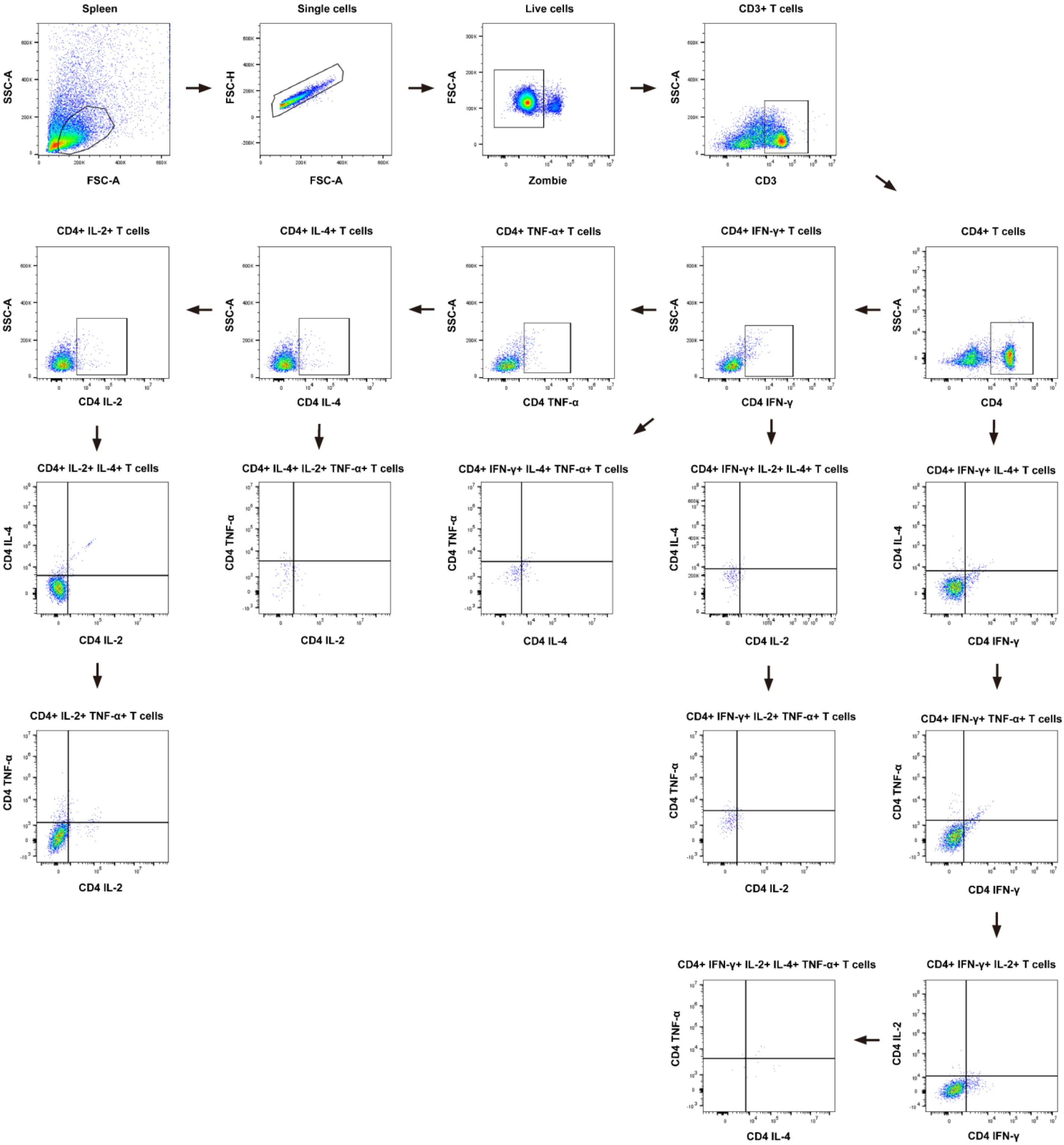

Figure S6. Flow cytometric gating strategies of expressing cytokines of CD4+ T cells.

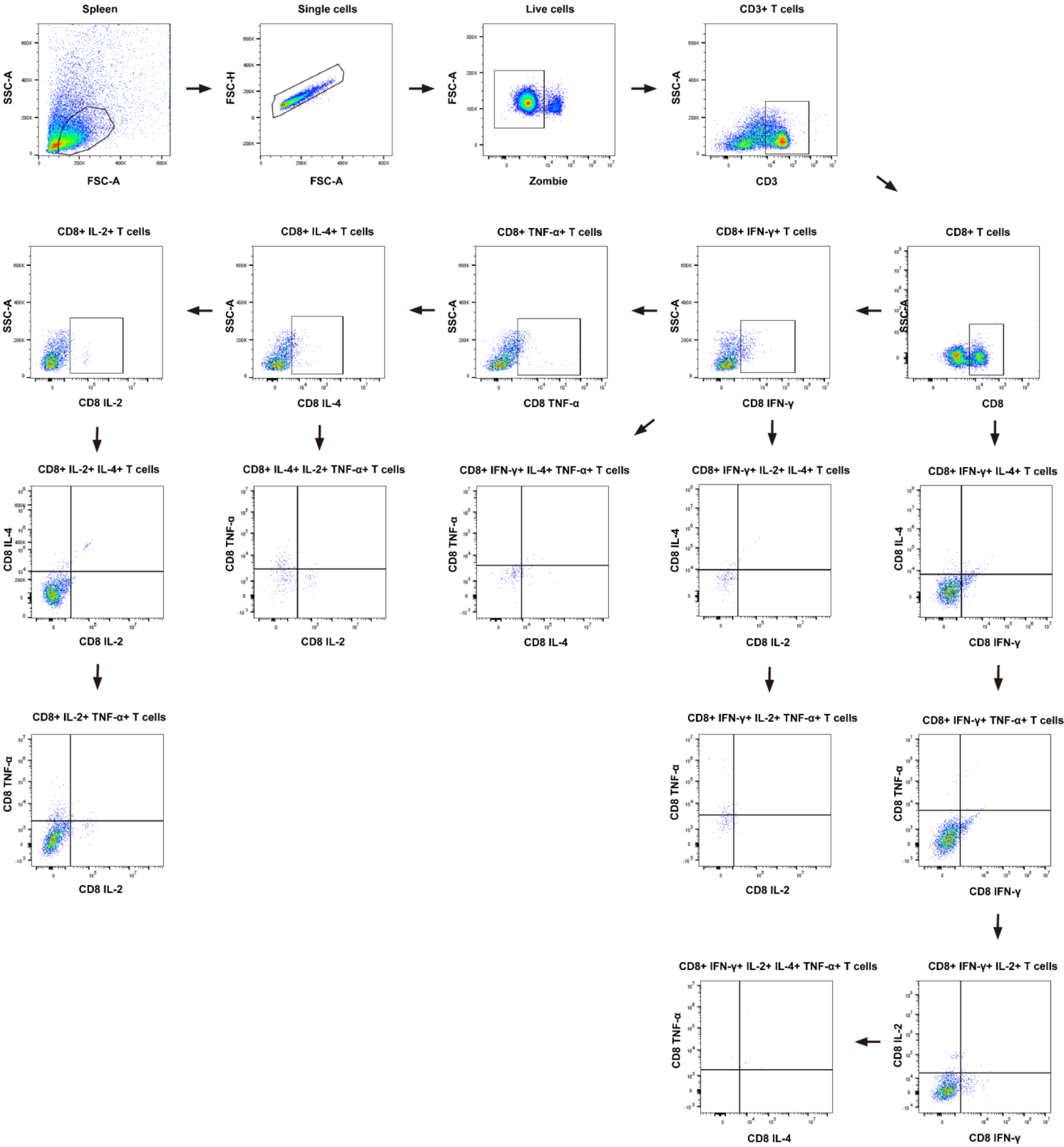

Figure S7. Flow cytometric gating strategies of expressing cytokines of CD8+ T cells.

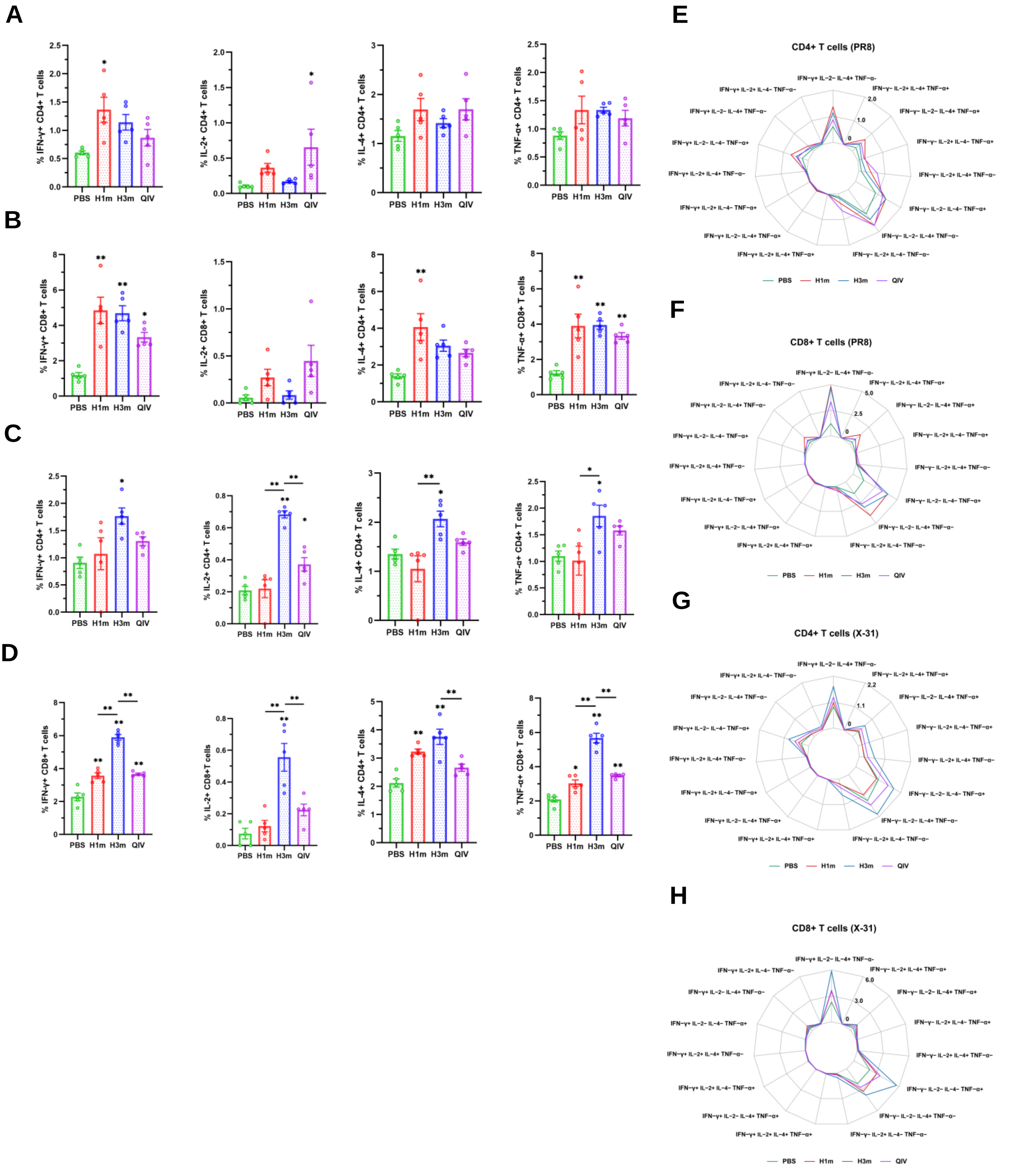

Figure S8. Recombinant mosaic HAs influenza vaccine immunization elicits T cell responses. BALB/c mice were vaccinated with Recombinant mosaic HAs influenza vaccine and compared to QIV-vaccinated mice. (A-D) Intracellular cytokine staining of splenocytes for (A, C) IFN-γ+, IL-2+, IL-4+, or TNF-α+ CD4+ T cells and (B, D) IFN-γ+, IL-2 +, IL-4+, or TNF-α+ CD8+ T cells following (A, B) A/Puerto Rico/8/34 (PR8) or (C, D) A/Aichi/2/1968 (X-31) stimulation. Data are presented as the mean with SEM (n = 5; one-way ANOVA with Tukey multiple comparison), *P < 0.05, **P < 0.01. (E-H) Radar plot shows polyfunctionality of the CD4+ or CD8+ T cell response. Geometric mean frequencies are displayed.

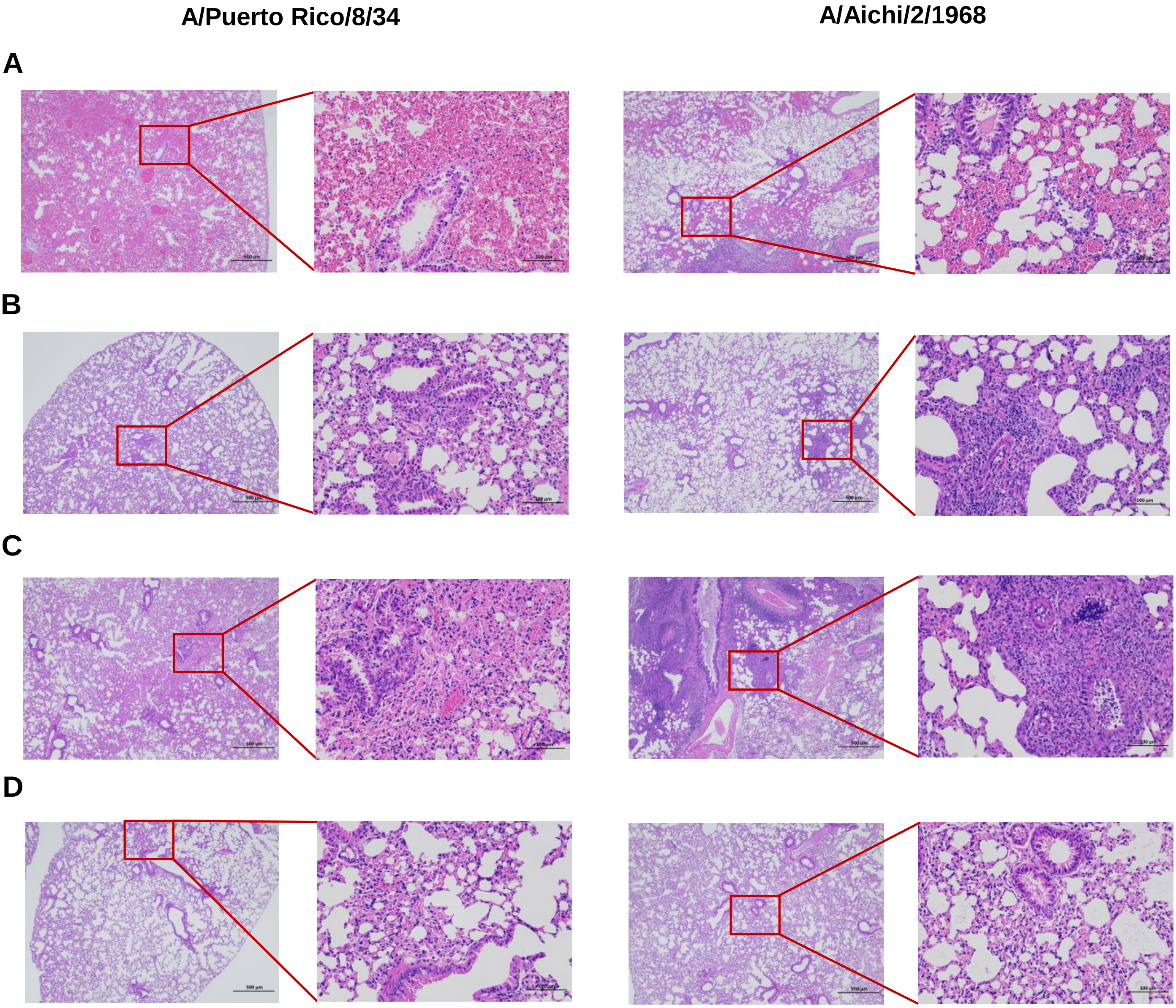

Figure S9. Lung histology of challenged mice. Mice were challenged with A/Puerto Rico/8/34 and A/Aichi/2/1968 viruses at week 5 following immunization with (A) PBS, (B) HAm, and (C) QIV. (D) Non-challenged control mice (Mock). At days 4 post-infection, mice were euthanized and lungs were harvested. Lungs were inflated with 10% formalin, fixed and processed for paraffin embedding, and 5-mm sections stained for H&E. Observations were made at 40× and 200× magnification.
